# A Newly Designed Fiber-Optic Based Earth Pressure Transducer with Adjustable Measurement Range

**DOI:** 10.3390/s18040932

**Published:** 2018-03-21

**Authors:** Hou-Zhen Wei, Dong-Sheng Xu, Qing-Shan Meng

**Affiliations:** 1 State Key Laboratory of Geomechanics and Geotechnical Engineering, Institute of Rock and Soil Mechanics, Chinese Academy of Sciences, Wuhan 430071, China; hzwei@whrsm.ac.cn; 2 School of Civil Engineering and Mechanics, Huazhong University of Science and Technology, 1037 Luoyu Rd., Wuhan 430074, China

**Keywords:** pressure transducer, soil pressure, sensor design, temperature compensation, Fiber Bragg grating

## Abstract

A novel fiber-optic based earth pressure sensor (FPS) with an adjustable measurement range and high sensitivity is developed to measure earth pressures for civil infrastructures. The new FPS combines a cantilever beam with fiber Bragg grating (FBG) sensors and a flexible membrane. Compared with a traditional pressure transducer with a dual diaphragm design, the proposed FPS has a larger measurement range and shows high accuracy. The working principles, parameter design, fabrication methods, and laboratory calibration tests are explained in this paper. A theoretical solution is derived to obtain the relationship between the applied pressure and strain of the FBG sensors. In addition, a finite element model is established to analyze the mechanical behavior of the membrane and the cantilever beam and thereby obtain optimal parameters. The cantilever beam is 40 mm long, 15 mm wide, and 1 mm thick. The whole FPS has a diameter of 100 mm and a thickness of 30 mm. The sensitivity of the FPS is 0.104 kPa/με. In addition, automatic temperature compensation can be achieved. The FPS’s sensitivity, physical properties, and response to applied pressure are extensively examined through modeling and experiments. The results show that the proposed FPS has numerous potential applications in soil pressure measurement.

## 1. Introduction

As much of the world’s infrastructure, such as high buildings, tunnels, dams, and subways, is built on soil, an understanding of soil pressure is essential to safety design and perform evaluation. Many disasters have occurred due to a lack of understanding of soil pressure during construction, such as the collapse of Nicoll Highway in Singapore on 20 April 2004 [1]. During the construction of the Mass Rapid Transit railway network between the Nicoll Highway and Boulevard stations, the cut and cover excavation collapsed, causing considerable damage [1]. The collapse occurred primarily because the field soil pressure exceeded the pressure provided by the supporting system. Soil pressure is also responsible for the long-term deformation, such as the settlement of the Pisa Tower, and caused a tunnel excavation collapse in Borràs Square, Spain [1]. These cases of geotechnical structural failure indicate that consideration of soil pressure is essential to design and construction. As soil is made up of particles, water, and air, pressure measurement in the soil mass is a critical task for civil engineers. 

Conventional pressure transducers based on electrical systems, micro-electro-mechanical systems (MEMS), and piezo-ceramic sensors are commercially available. The relevant electrical sensing technologies are electrical strain gauges or vibration wire strain gauges (VWSG), which have limited long-term stability and induce electromagnetic interference (EMI) [2,3]. MEMS and piezo-ceramic sensors are fairly well suited to dynamic pressure measurement; however, as soil pressure is normally static, they do not meet the requirements of soil pressure measurement. In recent decades, fiber optic sensors (FOS) have developed rapidly and show great potential for use in civil engineering. Compared with electrical sensors, FOS have many advantages, such as high accuracy and a high resolution, a tiny size, and resistance to EMI; even more importantly, the sensors can be connected into series in a single fiber [4,5]. Some researchers have used FOS to measure temperature [6], strain [7,8], deformation [9,10], water pressure [11], liquid level [12,13,14,15], humidity [16], and soil pressure [17]. Fiber Bragg grating (FBG) is one of the most widely used FOS technologies, due to its high accuracy and inexpensive data interrogation. For example, FBG has been used to measure soil small strain [18] and soil slope deformation [19,20].

The measurement accuracy and range are important criteria for civil engineering end-users. Researchers have made great efforts to improve the measurement accuracy. However, increasing measurement accuracy will decrease the measurement range. The most common configuration of soil pressure transducer is a flexible diaphragm with a steel chamber. A strain gauge or VWSG is glued to the surface of the flexible diaphragm to measure strain. A calibrated relationship is established between pressure applied to the diaphragm and strain in the diaphragm. As Wachman and Labuz [21] pointed out, soil arching may affect the output results. In addition, as the diaphragm of the pressure sensor is fixed at the boundary, the strain along the diaphragm is not uniform. A dual diaphragm design has been proposed to improve measurement accuracy [20,22]. This design is simple and easily fabricated. However, it has two limitations. First, the strain distribution on the surface of the diaphragm is highly non-uniform. This may affect measurement accuracy, as strain is measured as an average over the length of the strain gauge. Second, the diaphragm has limited deformation, which may restrict the measurement range of the earth pressure sensor.

In this study, to address the above-mentioned limitations, a new type of fiber optic based earth pressure sensor (FPS) for earth pressure measurement is proposed, fabricated, and analyzed. The working principle, fabrication details, and calibration tests are described. The newly designed FPS is carefully examined in laboratory tests. The results of these tests are presented and discussed, and the major findings highlighted.

## 2. Methodology

### 2.1. Principles of the FBG Sensor

**Figure 1:**
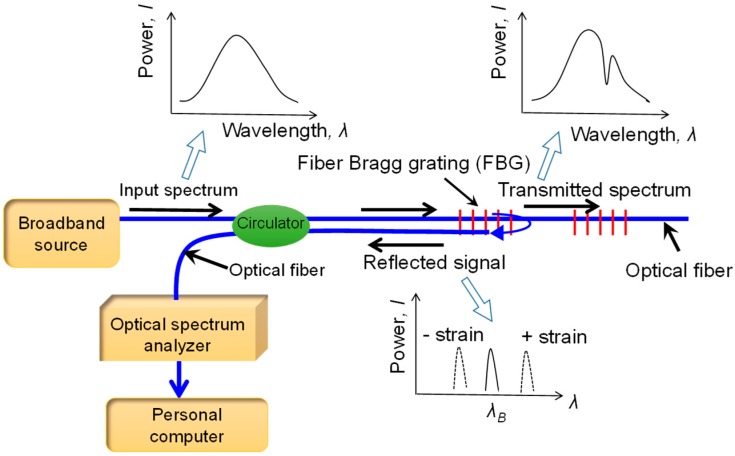
Working principle of the fiber Bragg grating (FBG) sensor.

Figure 1 shows the working principle of a FBG sensor. When broadband light is injected into the optical fiber, a signal will be reflected with a wavelength centered around a specific wavelength. The reflected wavelength is related to the physical properties of the Bragg sensor and can be expressed as [23]:

(1) $$\lambda_{B}=2n_{eff}\Lambda$$

where *λ_B_* is the reflected wavelength; *n_eff_* is the core index of refraction and Λ is the grating period of index modulation which are dependent on surrounding strains and temperatures. The wavelength shift (Δ*λ_B_*) has a relationship with the change in strain and temperature of the sensor, which can be determined by:

(2) $$\frac{\Delta \lambda_{B}}{\lambda_{B}}=\left[1-p_{e}\right]\Delta \varepsilon +\left(\alpha +\xi \right)\Delta T≅0.78\Delta \varepsilon +6.7\times 10^{-6}\Delta T$$

where *p_e_* is the elastic optical coefficient; *α* and *ξ* are the coefficients of temperature effect; Δ*ε* and Δ*T* are the change in strain and temperature, respectively. The FBG sensor developed in this study is fabricated using the phase mask method to write a special Bragg fiber into a single model fiber (SMF). Details of the phase mask method can be found in Xu [24]. This method is comprised of three steps: preparing a photosensitive fiber, creating a Bragg grating in the fiber using a phase mask, and thermal annealing. These processes permanently change the optical fiber, giving the specified Bragg wavelength. The diameter of the SMF is 900 μm. The length of the FBG ranges from around 5 mm to 10 mm.

### 2.2. Newly Designed FPS

**Figure 2:**
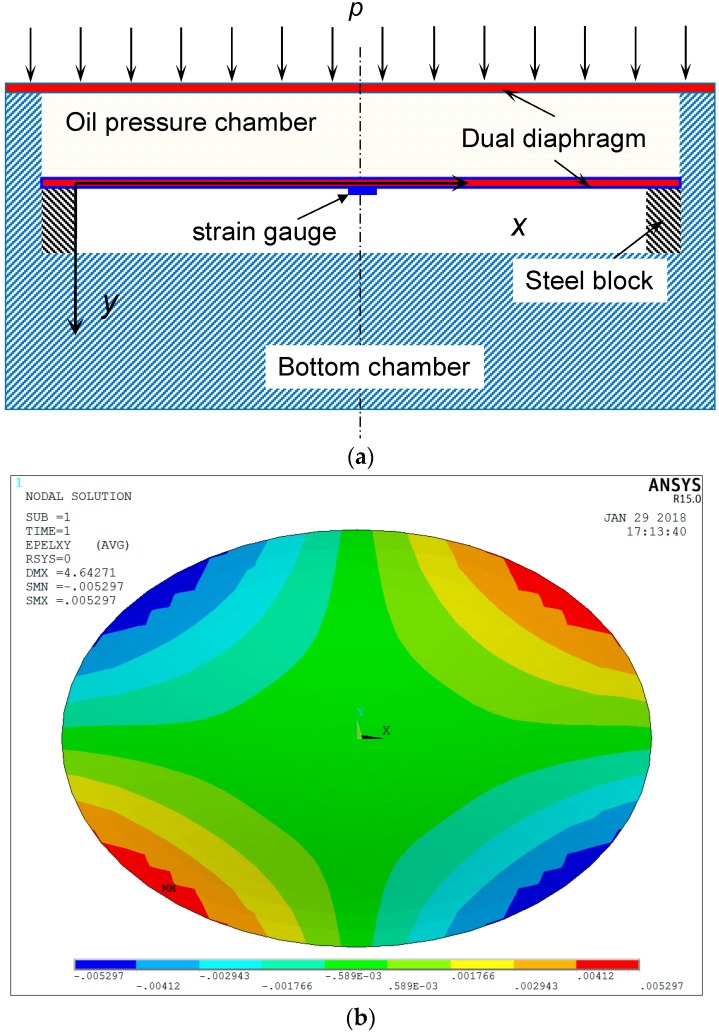
Earth pressure sensor with dual diaphragm design: (**a**) a traditional dual diaphragm design; (**b**) finite element analysis results of xy-shear strains of the diaphragm under normal pressure of 200 kPa.

Many earth pressure sensors used today are based on the dual diaphragm design as shown in Figure 2. The working principle of such sensors is the establishment of a linear relationship between the earth pressure applied to a diaphragm and strain in the diaphragm. The dual diaphragm design is simple and easy to fabricate. However, it has two limitations. First, the strain distribution on the surface of the dual diaphragm is highly non-uniform. Figure 2b shows the results of finite element model (FEM) analysis indicating that the shear strain on the surface of the dual diaphragm is non-uniform. This reduces measurement accuracy, as strain gauges, VWSG, and FBG sensors measure average strain over the length of a strain sensor. Second, the lower diaphragm has limited deflection, which may restrict the measurement range of the earth pressure sensor, as the deformation of the diaphragm is confined by boundary conditions. To overcome the above limitations, a new design for an FPS based on a combination of a diaphragm and a cantilever beam is developed and proposed in this study. The proposed method is expected to overcome the limitations of dual diaphragm FPS. It has the following advantages: (a) it provides automatic temperature compensation; (b) it is unaffected by the non-uniform strain distribution of the membrane; (c) it offers a larger measurement range than dual diaphragm FPS; and (d) its measurement accuracy is higher than that of the dual diaphragm approach.

**Figure 3:**
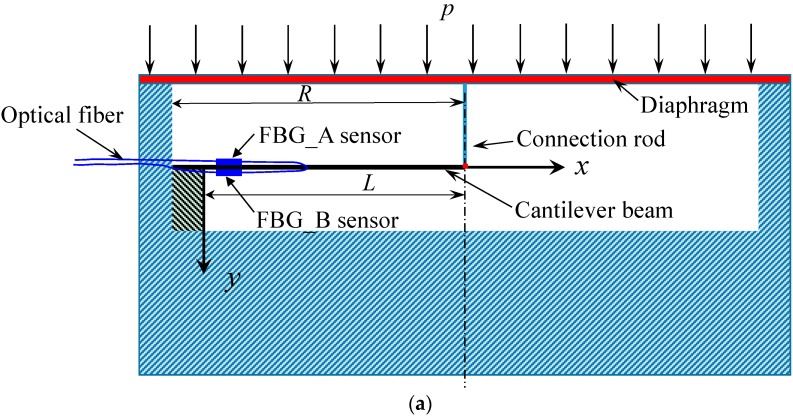

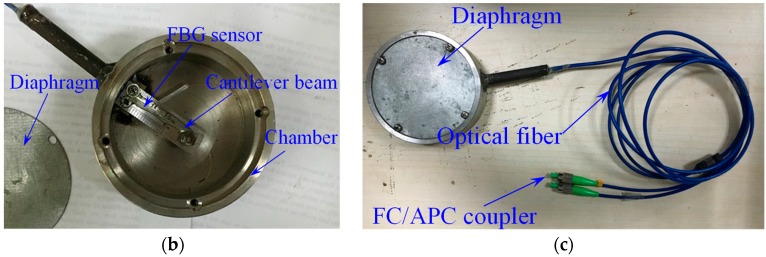
Schematic diagram of the fiber optic based earth pressure sensor (FPS) with combined diaphragm and cantilever beam: (**a**) schematic diagram of the newly designed FPS; (**b**) prototype of the designed FPS; (**c**) photograph of the packaged FPS.

Figure 3 provides a schematic diagram and photograph of the proposed FPS, which combines a diaphragm and a cantilever beam. The new FPS consists of a top cap diaphragm, a stainless-steel shell, a cantilever beam, a connecting rod, a base, two FBG sensors, external fiber cables, and screws. The working principle is as follows. Soil pressure is applied to the surface of the diaphragm, resulting in deflection. This deflection is transferred from the diaphragm to the cantilever beam, changing the strain exerted on the FBG sensors. The changes in strain on the FBG sensors are obtained using an optical interrogator and Equation (2). The relationship between the strain on the FBG sensors and the pressure applied is established as shown in the following section. In short, the pressure applied to the diaphragm is measured via the FBG sensors. Figure 3b,c show a prototype of the proposed FPS. The cantilever beam, FBG sensors, and connection rod are encapsulated in a 30 mm high steel chamber with a diameter of 100 mm.

### 2.3. Working Principles of the FPS

**Figure 4:**
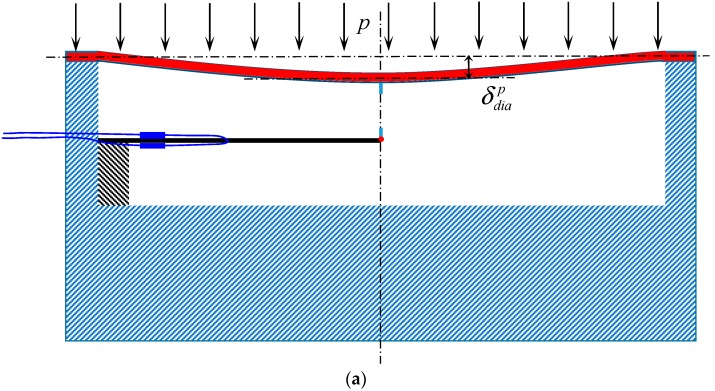

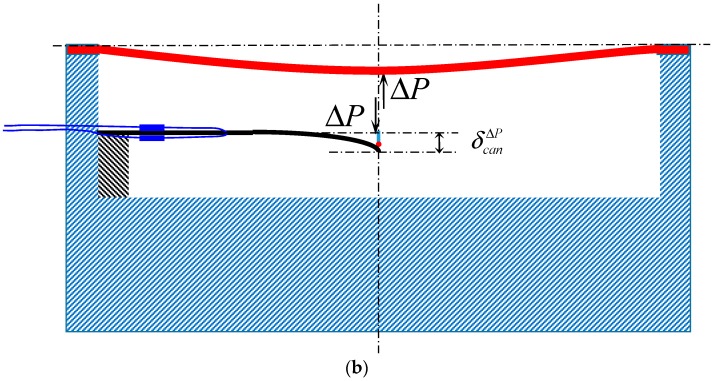
Working principles of the proposed FPS: (**a**) The deflection of diaphragm under an external force *p*; (**b**) the deflections of diaphragm and cantilever beam due to internal force ∆*P*.

The working principle is shown in Figure 4. As indicated in Figure 4a,b, the deflection at the center of the diaphragm *δ_dia_* can be divided into two parts: (a) deflection under a uniform pressure *p* applied on the surface of the diaphragm (Figure 4a and Equation (3a)); (b) deflection under an internal reaction force ∆*P* caused by the deformation of the cantilever beam (Figure 4b and Equation (3b)):

(3a) $$\delta_{dia}^{p}=\frac{pR^{4}}{64D}$$

(3b) $$\delta_{dia}^{\Delta p}=\frac{\left(-\Delta P\right)R^{2}}{16\pi D}$$

(3c) $$\delta_{dia}=\frac{pR^{4}}{64D}-\frac{\Delta PR^{2}}{16\pi D}$$

where *D = Et*^3^*/*12(1 *− μ*^2^), *p* is the pressure applied to the diaphragm, *E* and *μ* are the Young’s modulus and Poisson’s ratio, *t* and *R* are the thickness and radius of the diaphragm, ∆*P* is the internal reaction force applied reversely on the diaphragm. The deflection of the diaphragm will be equal to the deflection at the end of the cantilever beam because the rod is a rigid connection. Thus, the applied force imposed on the FBG-embedded cantilever beam, resulting in the bending strain can be expressed as follows:

(4) $$\varepsilon \left(x\right)=\frac{M\left(x\right)t_{c}/2}{E_{c}I_{c}}=\frac{\Delta P\left(L-x\right)\cdot t_{c}}{2E_{c}I_{c}}$$

where *x* is the location of the FBG sensor in a local *x*-coordinate system with the fixed end of the cantilever beam as its origin, *M*(*x*) is the change of the bending strain on the beam’s surface at *x*, *L* is the length of the beam, *E_c_* is the Young’s modulus of the beam, and *I_c_* is the moment of inertia of the cantilever beam with its width *b_c_* and its thickness *t_c_*. According to the Euler-Bernoulli beam theory, the maximum deflection of the cantilever beam Δ*d*_max_ due to the point force Δ*P* is obtained as:

(5) $$\Delta d_{\max}=\frac{\Delta PL^{3}}{3E_{c}I_{c}}$$

Substituting Equation (5) into Equation (4), we have

(6) $$\varepsilon \left(x\right)=\frac{3\Delta d_{\max}\left(L-x\right)\cdot t_{c}}{2L^{3}}$$

As mentioned before, deflection *δ_dia_* at the central portion of the diaphragm is the same as the maximum deflection of the cantilever beam Δ*d*_max_, thus the Δ*P* can be obtained as:

(7) $$\Delta p=\frac{pR^{4}}{64D}\cdot \frac{1}{\frac{L^{3}}{3E_{c}I_{c}}+\frac{R^{2}}{16\pi D}}$$

Considering Equations (5)–(7) simultaneously, the relationship between the strain measured by the FBG sensors, *ε*(*x*), and the force applied to the surface of the diaphragm, *p*, can be derived as follows:

(8) $$\varepsilon \left(x\right)=\frac{3\pi (L-x)t_{c}R^{4}}{8(\frac{4\pi EL^{3}t^{3}}{3(1-\mu^{2})}+3E_{c}I_{c}R^{2})}\cdot p$$

Automatic temperature compensation is realized as follows. When the cantilever beam is bent, the two *FBG* sensors (i.e., *FBG_A* and *FBG_B*) are used to obtain the strain on the cantilever beam, as indicated in Figure 4b. The strain measured has three components: bending strain, axial strain, and temperature strain. The axial strain and temperature strain are equal for both *FBG_A* and *FBG_B*. The bending strain values obtained for *FBG_A* and *FBG_B* have the opposite sign but the same absolute value. Thus, the bending strain of the cantilever beam can be obtained through Equation (9) by excluding the axial strain and temperature strain.

(9) $$\varepsilon_{B}=\frac{1}{2}\left(\varepsilon_{FBG\_A}-\varepsilon_{FBG\_B}\right)$$

where *ε_FBG_A_* and *ε_FBG_B_* are the measured strains of *FBG_A* and *FBG_B*. With this equation, the temperature effect can be eliminated. According to Equation (8), the pressure applied on the surface of the FPS can be obtained as:

(10) $$p=C_{2}\varepsilon_{B}$$

where $C^{2}=\frac{8(\frac{4\pi EL^{3}t^{3}}{3(1-\mu^{2})}+3E_{c}I_{c}R^{2})}{3\pi (L-x)t_{c}R^{4}}$ is the coefficient between the applied pressures on the FPS and bending strains measured by the FBG sensors.

## 3. Principle of Design and Optimization of Fabrication Geometry

The design of FPS should consider measurement range and resolution in detail. The measurement range and sensitivity of the proposed FPS depend on several parameters, such as the dimensions of the cantilever beam and the diaphragm, Young’s modulus for the beam and diaphragm, and the measurement range and sensitivity of the FBG sensors. The use of these parameters to ensure high sensitivity is examined in the section.

**Figure 5:**
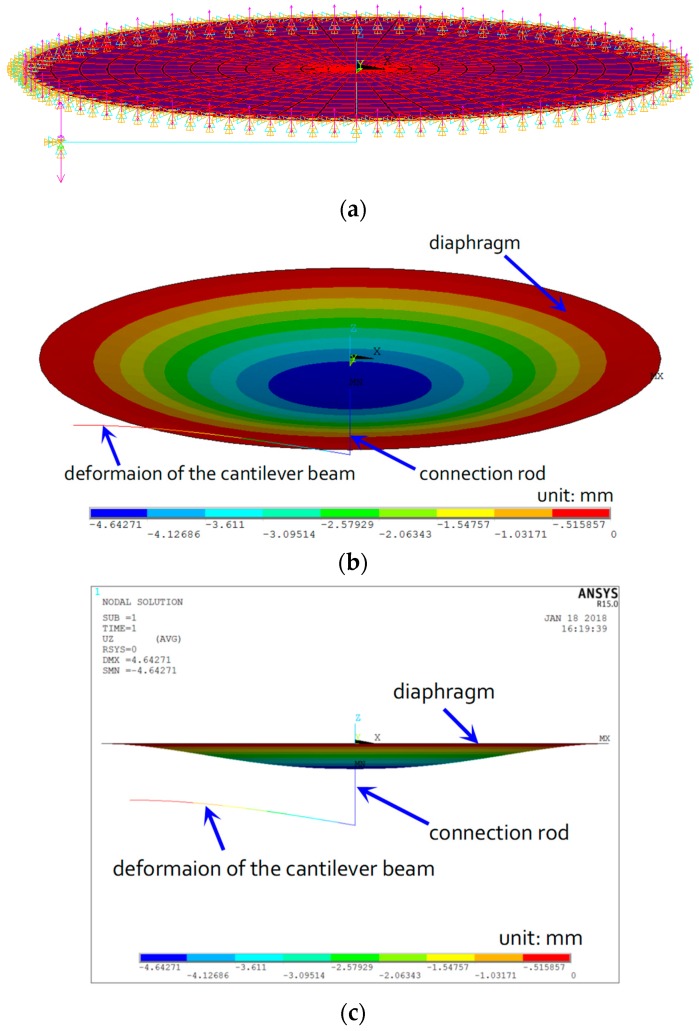
Simulation results of diaphragm and cantilever beam under external pressure of 200 kPa: (**a**) boundary conditions of the FEM model; (**b**) vertical deformation of the diaphragm; (**c**) vertical deformation of the diaphragm and the cantilever beam.

A finite element model (FEM) is established to analyze the stress and strain behavior of the proposed FPS by a commercial finite element software, ANSYS. The deflection of the diaphragm and cantilever beam under external pressure is analyzed with the FEM. The top cap of the FPS is simulated using a SHELL63 element. The cantilever beam is simulated using a beam element. The cantilever beam is 15 mm wide and 1 mm thick. Young’s modulus and Poisson’s ratio are 200 GPa and 0.3, respectively. The boundary conditions are indicated in Figure 5a. The outside of the diaphragm is fixed, and a stiff rod connects the diaphragm with the cantilever beam at the center of the model. The stiff rod transfers the deflection of the diaphragm membrane to the cantilever beam. An external pressure of 200 kPa is used to investigate the strain distribution on the diaphragm at different thicknesses. Figure 5b shows the results for axial strain on the diaphragm in the *x*-direction. As the strain is not uniformly distributed, directly measuring strain on the surface of the diaphragm would introduce errors. The unevenness of the strain distribution is clear from Figure 5c.

**Figure 6:**
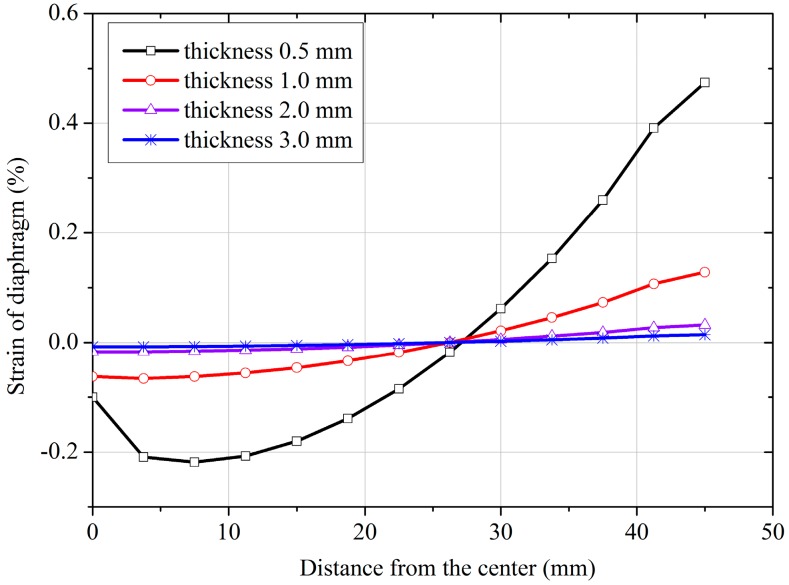
Simulation strain across the diaphragm for different thicknesses of the diaphragm.

The newly designed FPS overcomes the above-mentioned limitations by transferring the deflection of the membrane to the cantilever beam. FEM analysis is conducted to examine the diaphragm’s deflection behavior at different thicknesses. The simulation results are shown in Figure 6. The deflection at the center of the diaphragm increases as thickness decreases. Plastic strain is observed when the diaphragm is less than 1.0 mm thick. Plastic strain induces large measurement errors. However, when the thickness of the diaphragm exceeds 2.0 mm, the strain and deflection are too small, reducing the measurement accuracy of the cantilever beam. Thus, to obtain an accurate result, the thickness of the diaphragm should remain at around 1.0 mm.

The measurement range of the FPS depends on the maximum deflection of the diaphragm and the deflection of the cantilever beam. As pressure increases, the diaphragm is deformed, inducing the deflection of the cantilever beam. Suppose that the FBG sensors have a sensitivity of 1 micro-strain. The sensitivity of the FPS is defined as the variation in pressure under the strain on the FBG sensors, which is given in Equation (10).

**Figure 7:**
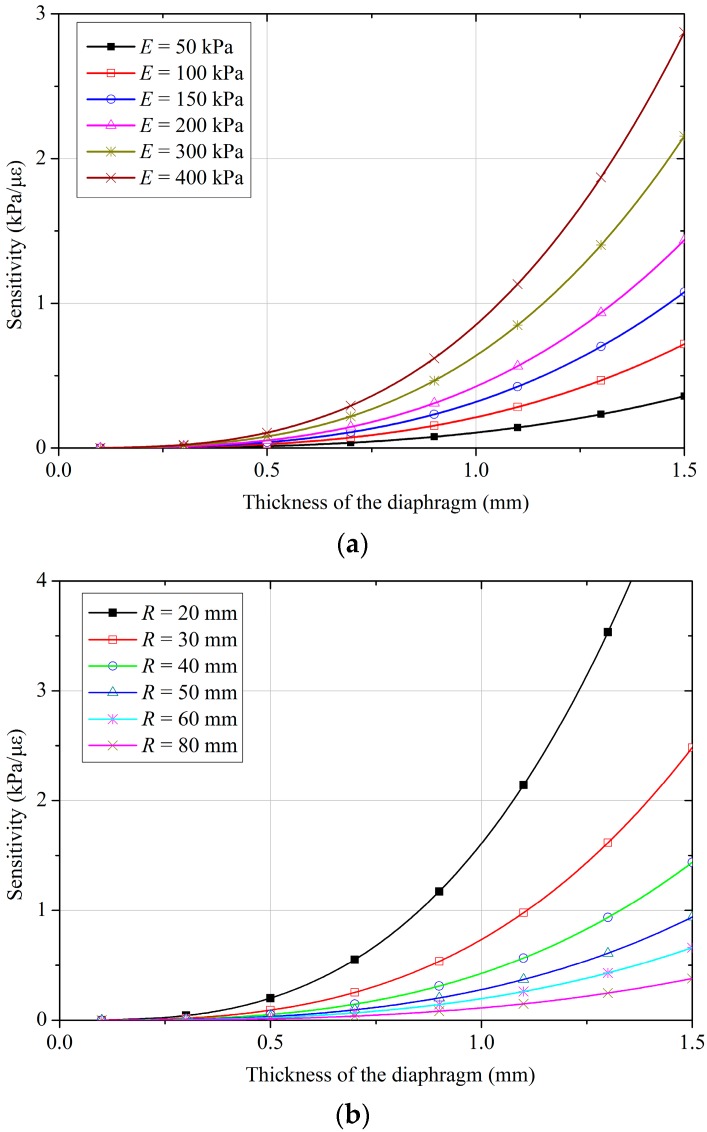
Sensitivity of the designed FPS with various thicknesses and (**a**) Young’s moduli, and (**b**) radii of the diaphragm.

**Figure 8:**
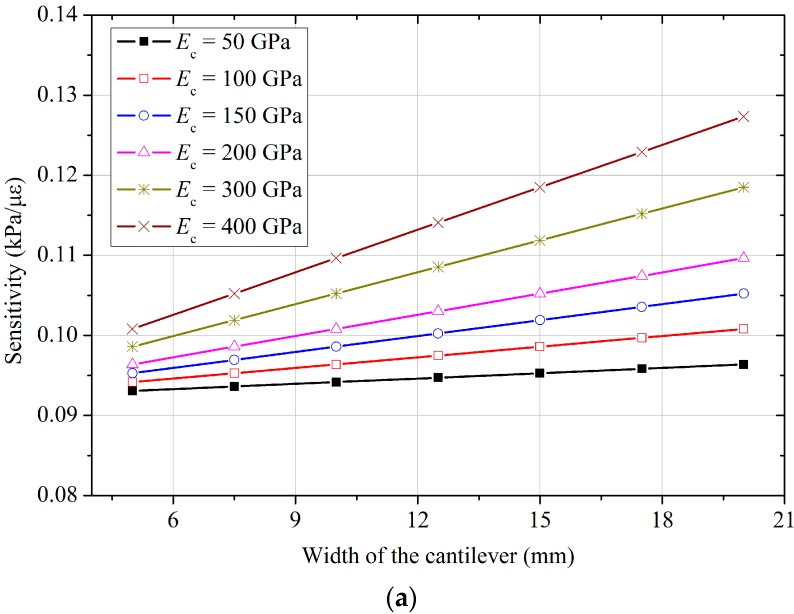

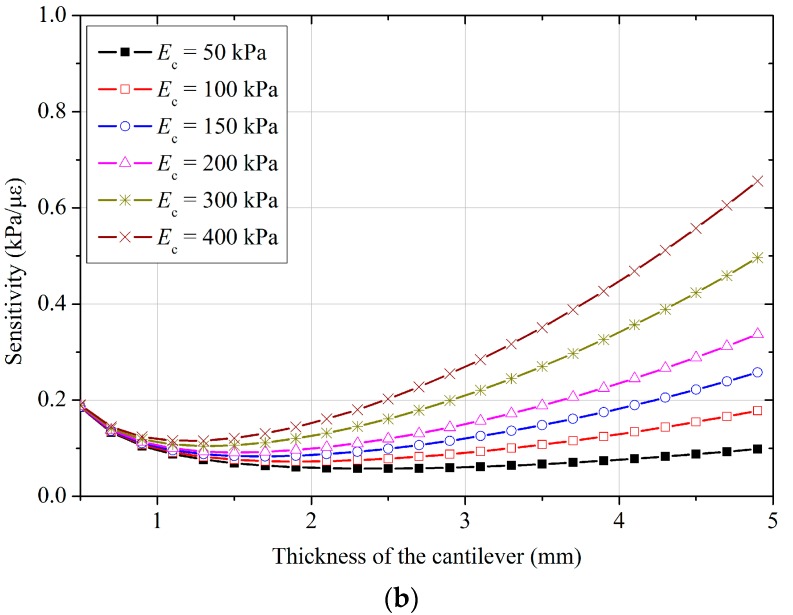
Sensitivity of the designed FPS with various Young’s modulus and (**a**) width, and (**b**) thickness of the cantilever.

A further parametric study is carried out to examine the effects of Young’s modulus for the diaphragm, diaphragm thickness, and the dimensions of the cantilever beam. Figure 7 shows the results of sensitivity analysis for different Young’s modulus values and diaphragm thicknesses, and Figure 8 shows how the sensitivity of the FPS varies with the width and thickness of the cantilever beam, respectively. The results indicate that the sensitivity of the FPS decreases with the diaphragm’s thickness and Young’s modulus, but increases with the radius of the diaphragm. For the cantilever-type FPS, a smaller Young’s modulus also yields a finer sensitivity, and sensitivity decreases linearly with the width of the cantilever. However, the difference is not significant between *b_c_* values of 5 to 20 mm. The relationship between sensitivity and cantilever thickness is not monotonic: the function initially decreases to its minimum value, then rises gradually with an increase in Young’s modulus for the cantilever. For a Young’s modulus of 200 GPa, the minimum sensitivity lies between cantilever thicknesses of 1 mm to 2 mm. Combining the above design principles, as indicated in Figure 3, a prototype of the FPS is designed and fabricated in the laboratory. The diaphragm of the FPS is made of a stainless-steel shell with a thickness of 0.6 mm and a diameter of 90 mm. The cantilever beam is also made of stainless steel, and is 40 mm long, 15 mm wide, and 1 mm thick. The FPS has an overall diameter of 100 mm and a thickness of 30 mm. As the FBG sensors used in this study have a sensitivity of 1.0 με, the sensitivity of the FPS as calculated using Equation (10) is 0.105 kPa/με.

## 4. Experimental Validation

**Figure 9:**
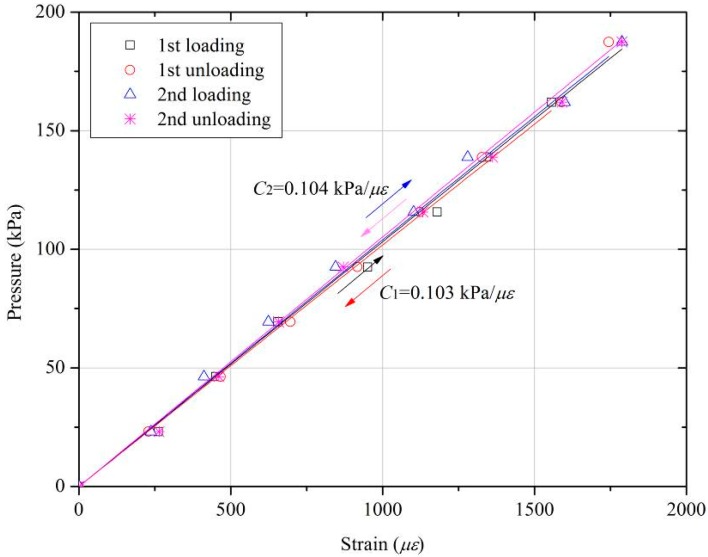
The calibration results of pressure-strain relationship between applied pressures and measured strains from FBG sensors.

An experimental validation test is conducted in the laboratory. Before performing each calibration test, the connecting screw is carefully checked to ensure that it touches both the top cap and the cantilever without creating initial deformation. A step load with different counterweights is applied to the surface of the FPS, and an FBG sensor interrogator is used to record the measurements taken by the FBG sensors. At each external pressure, the shift wavelength of the FBG sensors is measured using the sm125 Optical Sensing Interrogator from Micron Optics. Thus, the bending strain *ε_B_* of the cantilever beam can be calculated using Equations (2) and (9). Figure 9 shows the results for the applied pressure and bending strain calculated by the FBG sensors. Loading and unloading are repeated to confirm repeatability. The temperature effect is automatically eliminated using Equation (9).

The calibration results indicate that the proposed FPS responds well to changes in applied pressure. The strain on the FBG sensors is linearly related to the pressure applied. The fitting coefficients are 0.103 kPa/με (*R*^2^ = 0.9989) and 0.104 kPa/με (*R*^2^ = 0.9993) for cycle 1 and cycle 2, respectively. The standard fitting errors of the coefficient are 0.00121 and 9.34 × 10^−4^ for cycle 1 and cycle 2, respectively. Theoretical analysis (i.e., Equation (10)) yields a coefficient *C* of 0.1097 kPa/με for the proposed FPS for an FBG sensor strain sensitivity of 1.0 με*.* The coefficient obtained from experimental tests (i.e., 0.104 kPa/με) is quite close to the theoretical result.

Next, the newly developed FPS is compared with an FPS with the widely used dual diaphragm design. The response of the dual diaphragm pressure sensor is obtained from Equation (3a), and can be expressed as follows.

(11) ε=C⋅p

where $C=\frac{3\left(1-\mu^{2}\right)R^{2}}{8Et^{2}}$*. C* is the coefficient between the external pressure applied to the transducer and the strain on the diaphragm. The relative sensitivity of the two types of transducer is defined as *η* = *C*_2_/*C*_1_, where *C*_1_ indicates the coefficient of the dual diaphragm transducer, obtained from Equation (11), and *C*_2_ represents the coefficient of the newly designed FPS, obtained from Equation (10). To simplify the process of deduction, the following relationships are established between the parameters: *R* = *L*, *x* = 0, *b_c_* = 3/8*R*, and *μ* = 0.26. Then *η* can be simplified as follows.

(12) $$\eta =\frac{C_{2}}{C_{1}}=\frac{4t}{3t_{c}}+\frac{\left(1-\mu^{2}\right)}{4\pi }\cdot \frac{E_{c}}{E}\cdot \frac{b_{c}}{R}\cdot {\left(\frac{t_{c}}{t}\right)}^{2}$$

**Figure 10:**
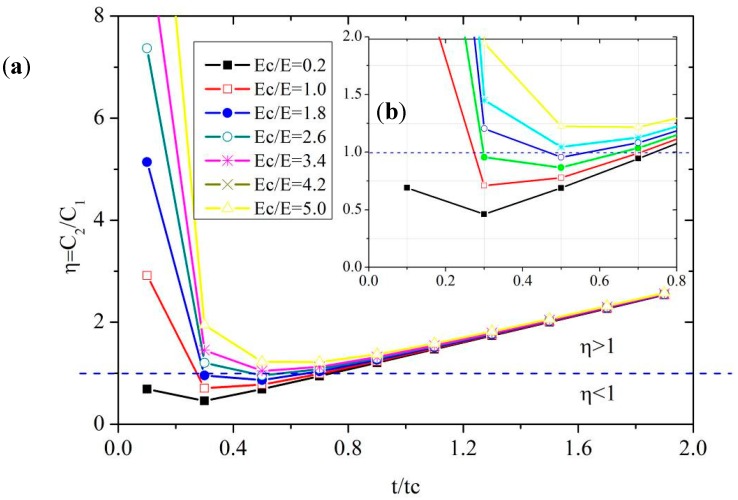
The relative sensitivity between cantilever and dual diaphragm type FPSs versus *t*/*t_c_* and *E_c_*/*E*: (**a**) a large-scale graph; (**b**) a small scale graph.

The results for *η* for *t*/*t_c_* values of 0 to 2 and *E_c_*/*E* values of 0 to 8 are presented in Figure 10. When *η* < 1 the proposed FPS is more sensitive than the dual diaphragm FPS. In this study, the material *E_c_*/*E* and *t*/*t_c_* values are 1 and 0.6, respectively. Thus, the sensitivity of the cantilever-type FPS is higher than that of the dual diaphragm pressure transducer.

## 5. Conclusions

The proposed FBG sensor is ideally suited for strain measurement. In this study, a new type of FPS is developed, fabricated, and verified, and its performance is analyzed in a series of laboratory tests. The major findings are as follows:

(1) The proposed FPS transducer, which combines a diaphragm with a cantilever beam, overcomes the limitations of the commonly used dual diaphragm design, as follows: (a) The measurement accuracy of the FPS is not affected by non-uniform strain distribution on the surface of the diaphragm; (b) a wider measurement range is provided; and (c) the measurement range can be adjusted by changing the parameters of the cantilever beam.
(2) The theoretical derivation and parametric studies of the proposed FPS reveal that the sensitivity of the cantilever-type FPS increases with the thickness, radius, and Young’s modulus of the diaphragm and the cantilever. A 1–2 mm thick cantilever beam yields an excellent sensitivity.
(3) A prototype of the proposed FPS is fabricated and verified in a series of laboratory tests. The new FPS transducer has a sensitivity of 0.104 kPa/με across applied pressures up to 180 kPa, and is thus more sensitive than the dual diaphragm type. Further analysis is performed to determine the *t*/*t*_c_ and *E*/*E*_c_ values and thus the relative sensitivity of the two designs, offering insights into a range of parameters required to fabricate an FPS transducer with even greater sensitivity for various applications.
